# Culture and community perceptions on diet for maternal and child health: a qualitative study in rural northern Ghana

**DOI:** 10.1186/s40795-021-00439-x

**Published:** 2021-07-15

**Authors:** Maxwell A. Dalaba, Engelbert A. Nonterah, Samuel T. Chatio, James K. Adoctor, Daniella Watson, Mary Barker, Kate A. Ward, Cornelius Debpuur

**Affiliations:** 1 Institute of Health Research, University of Health and Allied Sciences, Ho, Volta Region Ghana; 2 Navrongo Health Research Centre, Research and Development Division, Ghana Health Service, Navrongo, Ghana; 3 Julius Global Health, Julius Centre for Health Sciences and Primary Care, University Medical Centre Utrecht, Utrecht University, Utrecht, the Netherlands; 4 Global Health Research Institute, School of Human Development and Health, Faculty of Medicine, University of Southampton, Southampton, UK; 5 MRC Life Course Epidemiology Unit, University of Southampton, Southampton, UK; 6 NIHR Southampton Biomedical Research Centre, University Hospitals Southampton NHS Foundation Trust, Southampton, UK; 7 School of Public Health, Faculty of Health Sciences, University of the Witwatersrand, Johannesburg, South Africa; 8 School of Health Sciences, Faculty of Life and Environmental Sciences, University of Southampton, Southampton, UK

**Keywords:** Culture, Perceptions, Diet, Nutrition, Maternal and child health, Northern Ghana

## Abstract

**Background:**

This study explored cultural and community perceptions of optimal diet for maternal and child health in northern Ghana.

**Methods:**

This was an exploratory cross-sectional study using qualitative methods for data collection. Data were collected between March and April 2019 consisting of 10 focus group discussions with men and women community members between 18 and 50 years in the Kassena-Nankana districts of Ghana. Data were organised using QSR NVivo 12 qualitative software to facilitate thematic analysis.

**Results:**

All study participants recognised the importance of an optimal diet for mother, child and better pregnancy and breastfeeding outcomes. However, there were different cultural beliefs and taboos about what foods are healthy and non-healthy for women at different stages of the reproductive period. Foods perceived to be unhealthy for pregnant women were fatty foods and fresh meat (uncooked or unprocessed meat) due to the belief that they can lead to delivery complications, which many women feared. In addition, some participants relayed the cultural belief that pregnant woman should not eat eggs because it would make the child a thief. Lactating mothers are not to eat foods such as *vigna subterranean* known locally as bambara bean and *“gari”* (local meal made from cassava) because it is believed to inhibit breastmilk production. Participants emphasised that food insecurity and economic constraints meant women could not achieve optimal diet and could not afford to be selective in food choices.

**Conclusion:**

Community members recognized the importance of optimal nutrition but were constrained by poverty and cultural barriers. A dual approach which targets improvements of local food production and economic empowerment in combination with community-based discussion and education of the impacts of food taboos on health, should facilitate improvement in the diets of women and future generations.

## Background

Worldwide, improving maternal and child nutrition is a major public health challenge, and the situation is most severe in low and middle income countries [1]. Pregnant women and children under 5 years of age are most affected by malnutrition, which is a risk factor for morbidity and mortality both in childhood and into later life [1]. Nutritional status before conception, during pregnancy and in early life is associated with long-term health for mother and child [2].

In Ghana, undernutrition among pregnant women, breastfeeding mothers and children is highly pervasive and poses a huge challenge for the government. Although the Ghana government has introduced some policies and interventions such as the National Nutrition Policy (NNP), postharvest food storage techniques, and micronutrient supplementation to improve nutrition in the country, the impact has so far not been encouraging [3]. For instance, according to the 2014 Ghana Demographic and Health Survey, about 20% of children under 5 years were stunted in growth due to malnutrition [4] which can lead to impaired growth and development of the children.

There are also regional variations in the prevalence rates in undernutrition in Ghana, with the most affected regions being the northern regions. In Ghana, it was reported that in 2018, the prevalence rate of stunting was 37.1, 35.8, and 25.1% for the Northern, Upper East and Upper West regions respectively, while the prevalence of wasting was found to be 11.1, 11.2, and 7.3% in the same regions [3].

Consistent with these figures, there are high rates of poverty in northern Ghana when compared to the other parts of the country [5]. In addition to absolute issues of poverty and food insecurity in regions such as this, evidence from other low- and middle-income countries suggests that there are cultural beliefs about foods that also affect the diets and consequently nutrition status of mothers, pregnant women and children [6, 7]. Understanding what communities perceive to be appropriate diets for women and children, and what challenges they face in providing this diet is an important step towards developing interventions to address malnutrition in women and children. There are currently limited data describing these issues in northern Ghana. This study therefore explored community perceptions of what constituted an optimal diet for maternal and child health and barriers to providing this optimal diet in the Kassena-Nankana districts of rural northern Ghana.

## Methods

### Study area

The study was conducted in the Kassena-Nankana East and West Districts of the Upper East Region of northern Ghana. The Kassena-Nankana East Municipal is home to the Navrongo Health Research Centre (NHRC) and staff of the NHRC carried out the study. The NHRC operates a Health and Demographic Surveillance System (HDSS) which provides a data base of all individuals and households in the Kassena-Nankana East and West Districts.

The two districts cover an area of about 1675 km^2^ of land and the total population under surveillance is approximately 152,000 individuals residing in about 32,000 households [8]. The area is characterized by a rainy season spanning from May to October and a dry season from October to March. The people mainly live on subsistence farming with the main crops being millet, rice, maize and groundnuts. Vegetables such as tomato, pepper among others are also produced in the area. Many rural households’ rear cows, goats, sheep and poultry such as chicken and guinea fowl.

The districts are located in the region with the highest level of malnutrition compared to other parts of the country. The prevalence of stunting (a condition where children are too short in stature for their age and wasting (low weight-for-height) in the region is 22.4 and 9% respectively [4].

The districts have one referral hospital located in Navrongo town and eight health centres strategically located across the districts which provide secondary curative and preventive health care. There are 28 Community-based Health Planning and Services (CHPS) compounds/clinics located in various communities providing primary health care, treatment for minor ailments and also carrying out childhood immunizations and antenatal services. There are three private clinics, three pharmacies and over 50 licenced chemical /medicine shops in the area.

### Study design

The study was an exploratory cross-sectional qualitative study. All methods were carried out in accordance with qualitative guidelines and regulations [9]. Adult men and women from the community participated in focus group discussions (FGDs) between March and April 2019. A qualitative method was considered appropriate for this study given that it provides opportunity for FGD’s and to get a deep understanding of community perceptions of issues of interest [10].

This study was part of a larger study called the Improved Nutrition during Preconception, Pregnancy and Post-delivery project (INPreP project). The INPreP project is a National Institute of Health Research (NIHR) sponsored collaborative research project coordinated by the University of Southampton, which seeks to engage with community and relevant stakeholders in optimising nutrition in the 1000 days plus period and to prioritise solutions for Ghana, Burkina Faso and South Africa [11–14].

### Study population and sampling techniques

Study participants were community members in the Kassena-Nankana East and West Districts aged between 18 and 50 years. The Navrongo Health and Demographic Surveillance System (NHDSS) data base was used as the sampling frame for the selection of study participants. The NHDSS monitors births, deaths, pregnancies and other vital demographic events over a 120-day cycle. For purposes of this monitoring, the districts are divided into five zones: the East, West, North, South and Central zones. The East and South zones are predominantly Nankana ethnic groups while the West and North are Kassena ethnic with the Central zone having mixture of ethnic groups. These five zones are further sub-divided into clusters, compounds and households.

A simple random multistage sampling method was used to select participants for the FGDs. First, a simple random selection was used to select the south zone to represent the Nankana ethnic area and the north zone to represent the Kassena ethnic area. Since the Central zone is a cosmopolitan area, it was not included in the selection. A random sample of five clusters were then selected from each of the two selected zones.

Secondly, in each cluster, a list of 20 individuals who met the age, gender and ethnic criteria was generated from the cluster and targeted for participation in the FGDs. Data collectors then visited these individuals and the first 10 individuals in each category who were met were invited to participate in each FGD.

### Data collection procedure

The FGDs were conducted by experienced graduate level qualitative research officers in the relevant local languages. A total of 10 Focus Group Discussions (FGDs) were conducted. Before the data collection, the interviewers were trained on the research protocol and interview guide. A pre-test was conducted during the training to determine the appropriateness of the interview guide.

The FGDs with participants were organized separately based on gender (men, women), ethnicity (Kassena, Nankana) and further disaggregated by age (18–25 years, 26–39 years and 40–50 years for females; 24–34 years and 35–50 years for males). These groupings provided an opportunity to obtain varied views from women in different age groups across the reproductive period, men in the study area, and participants with two distinctively different ethnicities which were thought to potentially have different beliefs about food. Out of the 10 FGDs that were conducted, four were men and six women groups, which were conducted separately to ensure a more open and comfortable focus group dynamic (Table 1).

**Table 1:** Summary of Focus Groups and Participants

No.	Participants age group	Number of FGDs	Ethnicity	Gender	Number of participants (number per each group)
1	18–25 years	2	1 Kassena, 1 Nankana	Female	17 (8,9)
2	26–39 years	2	1 Kassena, 1 Nankana	Female	17 (9,8)
3	40–50 years	2	1 Kassena, 1 Nankana	Female	17 (9,8)
4	24–34 years	2	1 Kassena, 1 Nankana	Male	16 (8,8)
5	35–50 years	2	1 Kassena, 1 Nankana	Male	16 (8,8)
	**Total**	**10**			83

Discussions were held in the community using an interview guide. Each FGD was conducted by two research officers, one serving as a moderator and the other a notetaker, and interviews lasted about 1 h. The interview guide covered areas such as exploring optimal nutrition for mothers and children, nutritional challenges and potential solutions to improve nutrition.

### Data analysis techniques

FGDs were conducted in the two main local languages (Nankana or Kassena), audio recorded in the local language and later translated and transcribed into English. The transcripts were reviewed for accuracy and completeness and imported into QSR NVivo 12 qualitative software for coding. Guided by the objectives of the study and the themes from the transcripts, a code book was developed. Two independent researchers then coded the transcripts and thematic analysis carried out to identify and interpret themes or patterns in the data pertinent to the study aims [15].

## Results

### Socio-demographic characteristics of participants

A total of 83 participants took part in the FGDs with an average of eight per FGD (Table 1). Most participants (49.7%) were between 26 and 39 years, the majority (79.5%) were married, and 51.8% were from the Kassena ethnic group. About halve (50.6%) had primary/junior high education, and 44.6% were farmers. Thirty five percent (35%) of participants monthly earnings were between GH¢100 (18USD) and GH¢400 (70USD). About 50% of the study participants were unable to provide an estimate of their household monthly income (Table 2).

**Table 2:** Socio-demographic characteristics of participants

Variable	Frequency (***N*** = 83)	Percentage (%)
**Age**		
18–25	20	24.8
26–39	42	49.7
40+	21	25.5
**Sex**		
Male	32	38.6
Female	51	61.4
**Ethnicity**		
Kassena	43	51.8
Nankana	40	48.2
**Marital status**		
Never married	13	15.7
Married	66	79.5
Widowed	4	4.8
**Educational status**		
No education	22	26.5
Primary/ Junior high	42	50.6
Senior high/Tertiary	17	22.9
**Occupation**		
Unemployed	13	15.7
Civil/public servant	3	3.6
Self employed	30	36.1
Farmer	37	44.6
**Household monthly income (**GH¢**)**		
100–400	29	35.0
401–700	7	8.4
701+	5	6.0
Unable to tell	42	50.6

### Importance of pregnant and lactating women’s diet on the health of their infants

Both men and women admitted that an optimal diet is important for women during pregnancy, labour and breastfeeding as it leads to better health for the mother and child.

The lack of nutritious food was described as causing anaemia, underweight and general ill health in the mother and child as well as affecting child development. It was unanimously acknowledged by study participants that a malnourished or anaemic pregnant mother will not be able to gain the required health and vigour to function well and then have a safe and easy delivery. In addition, study participants mentioned that, when a lactating mother does not eat nutritious food, it will affect her health and ability to produce healthy and adequate breast milk for the baby. Some examples of statements by participants are given below:

*“Good nutrition helps the pregnant woman and the baby she is carrying to be strong and have enough blood. When a pregnant woman is eating nutritious food and she is about to deliver, it would be easy for her to deliver and she won’t have problems with blood (meaning to be anaemic). That is how I see it” (FGD-Females- 18-25 years).*

*“Eating well during pregnancy keeps the pregnant woman healthy and the baby she is carrying too gets food to eat. So, when she wakes up from bed without eating, she would give birth to a baby that is not strong and healthy, a sickling, a cripple, this too can affect the child’s brain. Hunger can cause all these things. So, eating well during pregnancy is good, it helps the pregnant woman, the unborn child; when she eats well, after delivering the child, the child wouldn’t be falling sick” (FGD-Males-24-34 years).*

*“When a woman is pregnant, eating well is very important; it helps to make delivery easy for her when it is time for her to deliver. The baby in her womb is healthy, when she gives birth the baby looks grown as if a year-old baby. This baby will look nice, and when people see this baby they would know that the mother actually ate good food before delivery. The woman looks strong and healthy, the baby she would deliver, the eyes will look bright, and there will not be dirt in the eyes” (FGD-Males-35-50 years).*

### Community perceptions of foods suitable for the health of pregnant women and lactating mothers

Various types of nutritious foods were perceived to be good for both pregnant women, particularly during delivery, and breastfeeding. They include soup made from leafy vegetables (kenaf leaves and dry baobab leaves), legumes/nuts (groundnuts and palm nuts) cooked with nutritious spices such as “dawadawa” (a local fermented decorticated African locust bean seeds) [16], low cost dried fish (dry Peeled Herring locally called “amani”), and shea butter used as cooking oil. Participants further mentioned that it is believed that if pregnant women eat the following staple, local foods regularly or in adequate quantities, they would be very healthy and during delivery they would not encounter any delivery complications and will give birth to a healthy baby.

The other solid foods or main meals that was mentioned were rice balls as well as local meals made from maize or millet flour such as “Tuo Zaafi” (TZ, a regular staple food in northern Ghana), [17, 18]. “Banku” (made from fermented corn, and usually orange size ball) [19]. and “pumpuka/ Kaponno” (porridge like meal made from millet) [20]. These soups and foods that is mentioned is reported to have essential nutrients and very good for general health and development [18, 20–23].

*“As a pregnant woman, you are supposed to eat vegetables and TZ to keep you healthy and you won’t have problems during labour” (FGD-Females-18-25).*

*“For a pregnant woman and breastfeeding mother, they have to eat “pumpuka” because it is nutritious. When she wakes up in the morning she should prepare “pumpuka” and eat …. They should also prepare Kenaf soup or groundnuts soup and eat with TZ or Banku. This will make them very healthy and be able to deliver a healthy baby” (FGD-Females-26-39 years).*

Some beverages were also recommended for breastfeeding mothers as it produce healthy breast milk. The majority of participants mentioned local foods such as “*Fura*” and “*Munnaa/zomkom*” (made from millet or sorghum flour) as nutritious meals/beverages for lactating mothers as it is perceived to increase breast milk to make a baby very healthy as well as improves the health of the mother. In addition, chewing fresh groundnuts and drinking Milo (hot chocolate drink with milk and micronutrients) were perceived to produce breast milk and therefore improves the health of the baby and mother.

*… we also have certain things they call “fura” (*made from millet dough) *that is good for women when they are breastfeeding. When a woman prepares this “fura” and takes it, it makes the breastmilk come more and it makes the child also gets satisfied and be healthy. So, I think such foods help the woman as well as the child (FGD-Male-24-34 years).*

*For a breast-feeding mother, to me, it is good for a woman to drink “munnaa” (this is processed flour from millet or guinea corn and added water to it) or fresh groundnut it helps to produce breast milk. That is what I have to say (FGD-Females-18-25 years).*

In addition, study participants mentioned that health workers informed them that to improve protein in the body, pregnant women are supposed to eat eggs and meat regularly. However, they expressed concern that accessing and purchasing eggs and meat, particularly in poor households, was a challenge due to poverty and financial constraints. They also commented that due to poverty they eat anything that is available as long as it is not bad that will cause gastro-intestinal problems (stomach upset, vomiting, diarrhoea). There is also belief from some that it is rather good to eat eggs and meat after delivery as it promotes recovery and good health to be able to produce healthy breast milk for the baby. In contrast, consumption of meat and eggs during pregnancy were perceived to lead to delivery complications.

*“To me, egg and meat is good for a pregnant woman for it gives them blood, it is not necessarily eating three or four eggs but eating one egg is enough. Within a week when you eat one, it is enough, here ‘we don’t have’ (we are poor) so if you get one egg within a week, it helps the baby to be strong and also makes you strong and also gives blood. Egg and meat are fine for the pregnant woman” (FGD-Female-26-39 years).*

“*… , that is why we earlier on said that poverty is the main issue. When you are pregnant, we know the foods that are good for us. But we don’t have the money to buy nutritious food or farm. Whatever we get, we eat, when you eat and you don’t vomit (if there is no stomach upset) then it is ok” (FGD-Women, 26–39 years).*

### Perceived type of nutritious foods suitable for children

All participants perceived that porridge (fluid-like beverage made from maize or millet) was the appropriate meal for children. They perceived that because of the age of the children they can easily take in porridge (since it is fluid-like) and it is also healthy for them. They however added that this could be taken alongside other solid foods. Study participants perceived that the complementary meals recommended for women mentioned above are not different for children between 1 and 5 years.

*When a child is 1 year, the appropriate food is porridge because it is easy to take and nutritious. Usually you are to prepare porridge for the child. In the morning when you prepare porridge for the child to take, in the afternoon and evening you can prepare vegetable soup with TZ for the child to eat. (FGD-Female-18-25 years).*

### Perceptions about unhealthy foods for pregnant women

Certain foods were perceived to be unsuitable or not healthy for pregnant women because they cause problems during delivery. Pregnancy cravings for baked solid white clay (Kaolin) [24] locally called *ferinkasa/Ayilo*, was mentioned by discussants as unhealthy for the pregnant woman and may cause delivery complications. Majority of the respondents mentioned that many pregnant women take this *ferinkasa* in the community*.* The reason given for them eating this baked solid white clay (*ferinkasa)* is that, it is perceived to help them tame diarrhoea, discomfort, nausea and other related pregnancy conditions. However, study participants mentioned that though their grandmothers (older people) and health workers have advised them to avoid *“ferinkasa”,* some pregnant women still take it.

*“… When you want to look, the pregnant women like eating unhealthy things like “ferinkasa” (white solid clay), but our mothers for instance the old women, they prevent them from eating these unhealthy things” (FGD-Female-26-39 years).*

Some study participants mentioned that pregnant women sometimes choose to avoid consuming fatty foods because they believe that it would make pregnant women gain weight and the fetus very large, leading to difficulties during delivery.

“… *now our women eat anyhow, she eats anything she gets. She is not selective; the baby in the womb becomes bigger, because of this when this baby is delivered you would think it’s a year born. This makes the delivery difficult for them that is the reason why most of them are operated. A pregnant woman should not eat fatty foods, in order to prevent the baby from growing big and to make the delivery easy for her” (FGD-Female-26-39 years).*

*“It is true when you eat the nutritious foods when you are about to deliver you will suffer. That is why they said we shouldn’t eat food that is nutritious but food that is not too nutritious so that we don’t have any problems” (FGD-Female-40-50 years).*

### Traditional/cultural beliefs about food unsuitable for pregnant women

Study participants mentioned that pregnant women are not to eat fresh meat because it will make the unborn baby to be big in the stomach which can result in delivery complications. The traditional belief that pregnant women should not eat fresh meat (uncooked or unprocessed meat), is an age-old belief that has been inherited and women are forced to adhere to it. It is perceived that fresh meat has a lot of protein that can make a pregnant woman and her unborn child gain weight. In contrast, they mentioned that pregnant women should rather eat smoked meat because it has low fat and low protein and is therefore deemed healthy.

*“… for a pregnant woman they tell you not to eat fresh meat, you don’t eat soup prepared with fresh meat. That if you eat fresh meat the child will become big in the stomach and so when you are about to deliver you cannot deliver” (FGD-Female-40-5- years).*

Similarly, women mentioned that pregnant woman are not to eat “sakotɔ” (the part of TZ that sticks to the bottom of the pot and become dry) because it is perceived that it makes the placenta grow big and causes delivery problems. “sakotɔ” is a common breakfast light meal eating in the northern part of the country.

The opinion regarding consumption of eggs and meat by pregnant women was not consistent among study participants. While some participants mentioned that eating of eggs and meat by pregnant women was good, the majority of the study participants mentioned that it was not appropriate for pregnant women to eat eggs and fresh meat because it makes the fetus big and cause difficult labour. It is also believed that if a pregnant woman eats eggs she would give birth to a child who will become a ‘thief’ when he/she grows.

*“… when a woman is pregnant you are not allowed to eat eggs because it would make the baby in the womb big and cause delivery problems” (FGD- Female-26-39 years).*

*“When a pregnant woman likes eating egg, she would give birth to a child who will become a thief” (FGD-Female-40-5- years).*

### Perceptions of types of foods not suitable for lactating mothers

Various foods were mentioned by study participants that they perceived unsuitable for breast feeding mothers as they tend to inhibit production of breast milk or contaminates the breast milk. Study participants mentioned “gari” as one food that is unsuitable for lactating women because it reduces the production of breast milk and also not nutritious. “Gari” is a dry-fried fermented cassava dough [19, 25] and in Ghana, it is perceived that “gari” is a low-cost meal and poor households usually depend on it for survival. “Gari” is less nutritious and contains mainly starch. In the study area, eating “gari” is common among households because of the poverty situation in the area.

*“What I want to say is that, gari is not suitable for a breast-feeding mother, because it has not got nutrients, it hasn’t got water; it is good for her to eat foods that contain water, so gari is not good for a breast-feeding mother” (FGD-Female-26-39 years).*

A few female participants held the view that breastfeeding mothers are not to eat bambara beans (Vigna subterranean) as it is perceived to be bad because it would produce bad breast milk. Bambara beans is a grain legume grown in sub-Saharan Africa and it is a meal frequently eaten in the study area. The study participants expressed that when a woman eats bambara beans and breast feeds a baby, the baby would have constipation.

*“Breastfeeding mother is not supposed to eat Bambara beans. If she eats the Bambara beans, when the baby wants to pass stool, it makes it difficult for him/her, the baby can’t pass stool. Even if you ignore the advice and still eat, the baby experiences stomach pains and begins to cry” (FGD- Female-26-39 years).*

## Discussion

The study explored community perceptions of optimal nutrition for maternal and child health as well as barriers or cultural beliefs about foods and eating habits of women in the Kassena-Nankana districts of rural northern Ghana.

All study participants recognised the importance of optimal nutrition to the health of the mother and child. The participants recognised that the lack of nutritious food can lead to anaemia, underweight as well as the general well-being of women who are pregnant, in labour and lactating. Different foods were perceived to be healthy and non-heathy depending on the woman’s reproductive period. For example, the study findings revealed that eating nutritious, more balanced diets including meat and eggs was believed to be very good for women particularly in promoting recovery and post-delivery health, including breast-milk production and its quality, but not believed to be good during pregnancy. These findings are in agreement of findings from previous study that reported that eating meat and eggs is recommended for women who have given birth [6].

Also, those who do not support the eating of fresh meat and eggs by pregnant women said it is a taboo that has been inherited from their ancestors. It is believed that when pregnant women eat fresh meat and eggs, they would gain weight and also make the unborn baby large which could lead to difficult labour. This is corroborated with other studies conducted in Africa that reported that pregnant women try to cut the intake of food such as meat and eggs to avoid labour difficulties [26–28]. In addition, it was mentioned that when a pregnant woman eats eggs and gives birth, the child will develop bad habit of stealing which is also reported in previous studies [25, 27, 29]. Though, it is important for pregnant women to have a balanced diet for themselves and the unborn baby [6], how much of weight gained or balanced diet considered to be “normal” in pregnancy needs further research. The vulnerable group (pregenant women, lactating mothers, and their childen) are therefore being deprived a valuable source of protein if they are prevented by taboos not to eat eggs and meat.

The main sources of nutrients in our study communities, particularly in rural areas and in poorer households, are from plant sources. However, one source of protein, consumed in the majority of household, is a low cost dried fish (locally called “amani”), which is usually obtained from the southern part of the country.

Generally, knowledge on the use of various plant and animal products forms a critical base for household dietary diversity for mother and child. Combinations of food can improve consumption and uptake of nutrients for a mother and child [26, 30]. However, food taboos and household economic resources restrict the intake of nutritious foods by pregnant, lactating mothers and children [11, 12].

It has been reported that food taboos can be found in virtually all human societies [27, 31]. Food taboos are set of rules or instructions from the forefathers/ancestors (God) that is being passed down from generations to protect community members from diseases [27]. Food taboos or cultural beliefs are generally meant to protect humans and promote good health. However, the fact is that these taboos mostly target the vulnerable group such as women, pregnant women and children. These food taboos spill out foods that are not to be eaten because it is perceived to have health consequences. These food taboos are usually based on casual explanation which can be supernatural, logical or sometimes hard to explain [31]. Community member’s belief noncompliance to taboos upsets the ancestors (gods) and this may result in harmful consequences from the ancestors. In the case of pregnant women, disobedience of taboos or ancestral laws may be associated to adverse pregnancy and delivery outcomes including death [27]. Therefore, women usually adhere to food taboos in the study area and it is also reported that, food taboos are respected and observed in all African countries [31, 32].

Food taboos can have positive or negative effects on humans [31]. With regards to nutrition, the positive effect is when the food taboo prevents people form eating harmful foods and the negative part is when the taboo prevents people from taking nutritious foods [27].

The baked solid white clay (Kaolin) [24] locally called *ferinkasa/Ayilo*, is a taboo for pregnant women in the study area which can be considered a positive effect of taboo. This “ferinkasa” that is mined in the depths of the earth contains chemicals such as Lead, Nickel and Arsenic as well as microorganisms such as *Bacillus, Pseudomonas, Mucor* and *Aspergillus spp* [24] which has negative effect on the human body and can lead to pregnancy complications and cancer [24]. Despite these few positive effects of food taboos, the negative effects of food taboos far outweigh the positive effects.

Some food taboos do not have scientific bases and can prevent people from eating healthy foods. Strategic education and discussion based on communities’s cultural beliefs from health workers with women, men and elders is needed to dispel some cultural beliefs on food taboos. Some of these foods such as eggs, bambara beans, meat that have been mentioned to be culturally unsuitable to eat by pregnant women are the common affordable foods with considerable amount of protein in th study community. Improvement in income generating activities would also help to ease the financial contraints to access to healthy food and general well being.

Although the government of Ghana has introduced a number of policies to improve nutrition in the country such as NNP, not much has been achieved over the period [25]. Infact, the goal of the NNP is to improve the nutritional status of the people, especially disadvantaged groups, including mothers, adolescent girls and children; to prevent and control malnutrition; and to accelerate national development through raising the standard of living [3].

There are however major implementation gaps in these policies due to multiple factors, resulting in suboptimal benefits to the target population in Ghana particularly northern Ghana [33]. The study district is located in one of the poorest regions (Upper East region) in northern Ghana [5, 34] and getting optimal nutrition is a challenge particularly in the rural poor households. The poverty situation in the study area is one of the main reasons for high undernutrition among pregnant women, breast feeding mothers and children in the area [3].

Generally, in Ghana most of the policies are progressive but the problem is usually in the implementation. Therefore, there is the need for engagements between health workers, community members and policy makers to strategize on the implementation of the NNP and other interventions in order to improve livelihood and nutrition in the country. Though health workers provide nutrition education and counselling to pregnant women during antenatal care visits, the effect is not much, given that some women still abide by their traditional beliefs or food taboos. This therefore suggests that health workers need refresher trainings on nutrition and communication that will incorporate some of these beliefs or food taboos in the education. Also, community elders should also be engaged in the nutrition education since they are the custodians of the land and taboos and therefore have influence. Furthermore, health workers should be resourced both financially and with adequate knowledge and logistics to enable them to provide nutrition education to community members.

### Strengths and limitations of the study

Interviews were conducted in the local languages of the study area and translated into English for analysis. It is possible that the real meaning of some statements made in the local languages may have been lost in the English translation. Nevertheless, the interviews were translated and transcribed by experienced research officers who are natives of the area and hence, the misinterpretation of the statements or words made in the interviews would be minimal and may not affect the results of the study.

Even though the interviewers tried to obtain the monthly income of households, about half of FGD participants were unable to provide an estimate of their household monthly income. We cannot be sure that this did not impact the results of the findings of this study, but as all participants were drawn from similar communities, it is unlikely to intrinsically change the findings.

Responses of study participants could also influence the study findings particularly on the traditional/cultural barriers of some foods, given that they know that the study team are from the health sector and are usually against taboos. However, the study team were well trained to allay the fears of participants to provide accurate responses that reflect what patterns in the community.

## Conclusions

The importance of an optimal diet for maternal and child health is understood in these communities, but opportunities for women to eat in this way are severely constrained by food insecurity and poverty and are further shaped by culturally-defined taboos. A dual focus on improving local food production and economic empowerment in combination with community-based discussion and education of the impacts of food taboos on health will facilitate improvement in the diets of women and future generations.

## Data Availability

Relevant data based on which conclusions were made are included in the document.

## Abbreviations

CHPS: Community Based Health Planning and Services
ERC: Ethical Review Committee
FGD: Focus Group Discussions
HDSS: Demographic Surveillance System
HDSS: Navrongo Health and Demographic Surveillance System
NHRC: Navrongo Health Research Centre
NIHR: National Institute for Health Research
ODA: Official Development Assistance
